# Highly Reproducible, Bio‐Based Slab Photonic Crystals Grown by Diatoms

**DOI:** 10.1002/advs.201903726

**Published:** 2020-03-21

**Authors:** Johannes W. Goessling, William P. Wardley, Martin Lopez‐Garcia

**Affiliations:** ^1^ International Iberian Nanotechnology Laboratory Braga 4715‐330 Portugal

**Keywords:** bioinspiration, diatoms, frustules, nanofabrication, photonic crystal slabs

## Abstract

Slab photonic crystals (PhCs) are photonic structures used in many modern optical technologies. Fabrication of these components is costly and usually involves eco‐unfriendly methods, requiring modern nanofabrication techniques and cleanroom facilities. This work describes that diatom microalgae evolved elaborate and highly reproducible slab PhCs in the girdle, a part of their silicon dioxide exoskeletons. Under natural conditions in water, the girdle of the centric diatom *Coscinodiscus granii* shows a well‐defined optical pseudogap for modes in the near‐infrared (NIR). This pseudogap shows dispersion toward the visible spectral range when light is incident at larger angles, eventually facilitating in‐plane propagation for modes in the green spectral range. The optical features can be modulated with refractive index contrast. The unit cell period, a critical factor controlling the pseudogap, is highly preserved within individuals of a long‐term cultivated inbred line and between at least four different *C. granii* cell culture strains tested in this study. Other diatoms present similar unit cell morphologies with various periods. Diatoms thereby offer a wide range of PhC structures, reproducible and equipped with well‐defined properties, possibly covering the entire UV‐vis–NIR spectral range. Diatoms therefore offer an alternative as cost‐effective and environmentally friendly produced photonic materials.

## Graphical abstract

This work demonstrates that diatom microalgae evolved silicate girdles with precise lattices reminiscent of 2.5D slab photonic crystals. Optical measurements show highly reproducible bandgaps and waveguiding in the visible/near‐infrared. While production of such advanced photonic materials usually involves modern nanofabrication technologies and elaborate cleanroom facilities, diatoms grow these structures requiring only water, nutrients, and light.

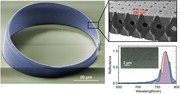

## 1 Introduction

Photonic crystals (PhCs)—nanostructures with periodic features on the wavelength scale of light—have a wide range of applications in optical technologies, including high power lasers and quantum logic devices.^[^ ^1^, ^2^, ^3^ ^]^ Recent observations confirmed that PhCs with different functionalities evolved in nature, where they have been described for different biological phyla within the animal kingdom, foremost in invertebrates^[^ ^4^ ^]^ and some vertebrates.^[^ ^5^ ^]^ While PhCs in the animal kingdom have been mostly attributed to functions related to color production,^[^ ^4^ ^]^ some recent discoveries demonstrated the presence of PhCs with light harvesting functions in photosynthetic organisms. These findings include vascular plants^[^ ^6^ ^]^ and macroalgae^[^ ^7^ ^]^ suggesting a widespread natural functionality to manipulate light for light harvesting through nanostructuring.

PhCs are usually formed by a periodic structure at wavelength scale with high refractive index contrast to the bulk material. Since the patterning induces wave interference, the optical properties of PhCs are strongly coupled to their morphologies.^[^ ^8^ ^]^ After the detailed investigation of PhCs over the last two decades, researchers identified a full zoo of structures (both natural and artificial) with a variety of exotic optical properties. Natural photonic systems, as described to date, typically feature 1D multilayer structures^[^ ^9^ ^]^ or complex 3D photonic structures.^[^ ^10^ ^]^ In many cases, such morphologies have been a source of bioinspiration for artificial structures with similar functionalities using technologically relevant materials.^[^ ^11^ ^]^ From a technological point of view, some of the most advanced PhCs are those referred to as slab PhCs (sPhCs).^[^ ^12^ ^]^ They consist of micron‐thick slabs of a dielectric material over which a periodic structure with different refractive index is patterned. As the slab acts as a waveguide, the periodic nanostructure can modify light propagation, causing photonic band gaps, i.e., directions for which light of a given wavelength cannot propagate. The sPhCs are increasingly common in cutting edge technologies, ranging from low footprint lasers^[^ ^13^ ^]^ to quantum optics,^[^ ^14^ ^]^ but such sPhC have not been observed in nature.

One of the natural structures more commonly suggested to present PhC properties evolved in diatoms. These globally abundant microalgae feature a unique structure called the frustule; an inorganic exoskeleton made of silicon dioxide (SiO_2_) encapsulating the diatom cell. Frustules have been investigated due to their highly ordered porous network, suggesting PhC properties in some species.^[^ ^15^, ^16^ ^]^ However, despite several studies that investigated the optical properties of frustules in different diatoms, there has been no clear experimental evidence of the frustule PhC functionalities. Since the frustule is unique to diatoms, it was proposed that this structure could have influenced their global abundance and species diversification.^[^ ^17^ ^]^ However, potential biological functionality of the frustule for the organism is still hotly debated and remains elusive.^[^ ^18^ ^]^ One hypothesized function is the protection of the cell against micro‐grazers, as the frustule provides high mechanical strength combined with low Young´s modulus of elasticity.^[^ ^19^ ^]^ The frustule also facilitates cellular conversion of bicarbonate to CO_2_, promoting photosynthesis in aquatic environments by acting as a diffusion barrier.^[^ ^20^ ^]^ The pores allow for chemical communication with the environment, but may simultaneously prevent harmful agents like bacteria or viruses from entering the cell via size exclusion.^[^ ^21^ ^]^ Furthermore, as the period of the structures have dimensions that facilitate interaction with photosynthetically useful sunlight, there is significant speculation that the ultimate function of frustule perforation is modulating light‐cell different interactions, e.g., by acting as a diffraction grating^[^ ^22^ ^]^ or as a PhC.^[^ ^16^ ^]^

**Figure 1:**
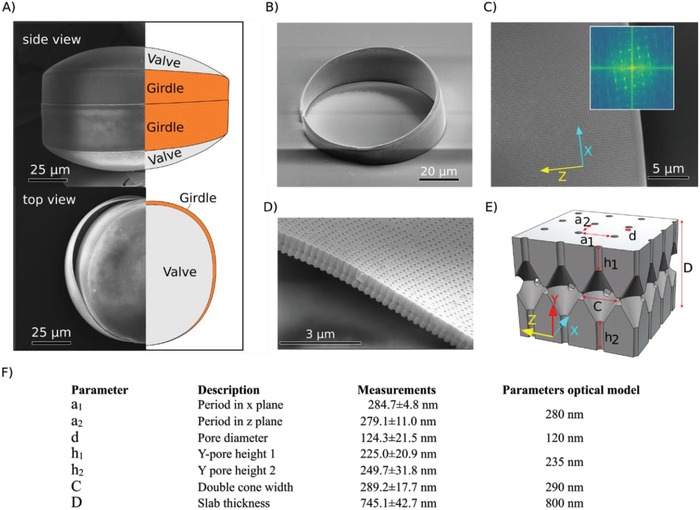
Frustule and girdle internal structure in the diatom *Coscinodiscus granii*. The frustule of this species contains four SiO_2_ parts, i.e., two valves and two girdles. A) The girdles encircle the valves at the overlapping regions keeping the frustule together. Intact frustule from side and from top view, with a girdle band visibly separating on the left side. B) Overview demonstrating the hollow cylinder character of the SiO_2_ girdle and the split ring spacing (on the left side). C) Surface micropores of the girdle in square lattice arrangement. Inset: Fast Fourier transform analysis of the lattice from the micrograph, demonstrating high periodicity of the pore arrangement. D) Cross‐section showing internal structure of the girdle along the *Z*‐axis. E) CAD reconstruction of the girdle crystal structure over four unit cells. Letters indicate the lattice parameter used in the optical model. F) Unit cell characterization of the *C. granii* girdle. Measurements were performed on SEM micrographs, representing measurements of five individual girdles of the diatom *C. granii* (strain K‐1834). Periods (*a*_1_ and *a*_2_), as well as pore diameter (*d*), were determined on ten individual SEM images of the same diatom strain. Parameters used in the optical models are indicated.

The two main morphological categories of diatoms are the centrics (subject of this study), which are round and show radial symmetry, and the pennate diatoms, which are elongated with lateral symmetrical frustules. The frustule can be simplified as a construct made of different silicate pieces: i) two convex‐shaped structures named valves and ii) two (or more) belt‐like structures named girdles, located at the overlapping regions of the valves (**Figure** **1**A).

Regardless of its potential morphological importance, the girdle is a largely understudied part of the frustule in terms of its optical properties, as almost all experiments concerned the diffractive effects of the valve. In the few studies observing the girdle optical properties, this frustule piece has been described as a structure formed by a square lattice of cylindrical holes perforating a SiO_2_ slab.^[^ ^16^, ^23^ ^]^ The photonic properties of the girdle were previously only theoretically investigated and described as sPhCs,^[^ ^16^ ^]^ where the dispersion is tailored by the photonic environment to form photonic bands.^[^ ^12^ ^]^ To date there was no experimental evidence of the sPhC nature of the diatom frustule.

We here demonstrate that the girdle of the diatom *Coscinodiscus granii* is a well‐defined and highly preserved sPhC. We also demonstrate that the nanometric morphological features are highly reproducible under laboratory growth conditions, which unveils great potential for technological applications where fabrication up‐scaling might be necessary. In this paper, we first show a comprehensive morphological and material characterization of the girdle 3D nanostructure by using scanning electron microscopy (SEM) techniques. Next, we present the optical properties, obtained using an adapted microscatterometry setup (see earlier description in ref. [6]), to measure the photonic properties in the in‐plane direction, ensuring coupling to the confined guided modes, as would be expected for sPhCs.^[^ ^24^ ^]^ Using focused white light illumination, we demonstrate in‐plane waveguiding, facilitating high symmetry directions of the PhC (Figure S1, Supporting Information). Finally, we discuss the well‐defined sPhC properties in light of evolutionary pressure and the potential of such reproducibility for industrial exploitation.

## 2 Results

### 2.1 Crystal Morphology and Material Properties of the Girdle

Figure 1B shows an example of the girdle of the diatom *C. granii* studied here. The girdle is a circular silica slab, slant cut toward a split ring spacing. The radius of the slab depends on the cell diameter, which can vary from 40 to 200 µm in the species *C. granii*.^[^ ^25^ ^]^ The height of the slab within one individual girdle differs, as it tapers toward the split ring space. Measurements over SEM micrographs show that the girdle band thickness was *D* ≈ 745.1 ± 42.7 nm. These values contrast with previous assumptions about the photonic properties of the girdle in diatoms.^[^ ^16^ ^]^ Fast Fourier transform analysis of SEM micrographs confirmed the well‐defined square lattice of micropores (Figure 1C), with similar period (*P* = 0.257; *N* = 10) along the *X*‐ and *Z*‐direction, i.e., *a*_1_ = 284.7 ± 4.8 nm and *a*_2_ = 279 ± 11.0 nm, respectively. A set of cylindrical micropores in the *X*‐ and *Z*‐directions intersects a central rhombic chamber, with the micropores inter‐connected along the entire girdle slab (Figure 1D/E). Based on the structural dimensions of the unit cell defined in the table shown in Figure 1F, we calculated that the total volume occupied by micropores and rhombic chambers can account for ≈25–30% in the unit cell (see the Experimental Section). This volume defines the void filled with the surrounding medium, causing refractive index contrast.

**Figure 2:**
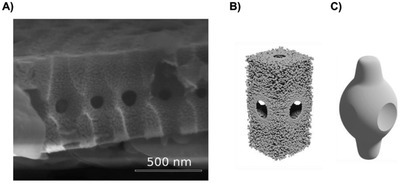
Micro‐ and nanoporosity defining the void filling volume in the *C. granii* girdle. A) SEM micrograph of a girdle sliced along the *Z*‐axis. The micrograph indicates nanoporous characteristics of the girdle. B) CAD illustration of the unit cell including nanoporosity (δ_i_ = 0.05). C) CAD illustration demonstrating the void presented by micropores.

In addition to the refractive index contrast between micropores and silica, the material properties of the silica slab will also affect the photonic characteristics. Earlier studies assumed a constant refractive index of the silica slab, usually *n* = 1.43.^[^ ^15^, ^16^ ^]^ However, it has been communicated that the frustule could feature nanoporosity.^[^ ^26^ ^]^ The girdle would therefore have a combination of two types of pores: the micropores that form the periodic structure and, in addition, nanopores that contribute to the material properties of the silica slab. **Figure** **2**A shows an SEM micrograph where such nanoporosity can be appreciated, followed by a sketch of a girdle unit cell, illustrating these two different pore types (Figure 2B). The aspect is important for the optical description, because nanoporosity influences the effective refractive index of the silica slab, as the surrounding medium could penetrate into the nanopores.^[^ ^27^ ^]^ We address this important aspect during the following paragraphs in more detail, when measurements and modeling of the girdle photonic properties are presented.

### 2.2 Photonic Properties by Experimental Means and Simulations

**Figure 3:**
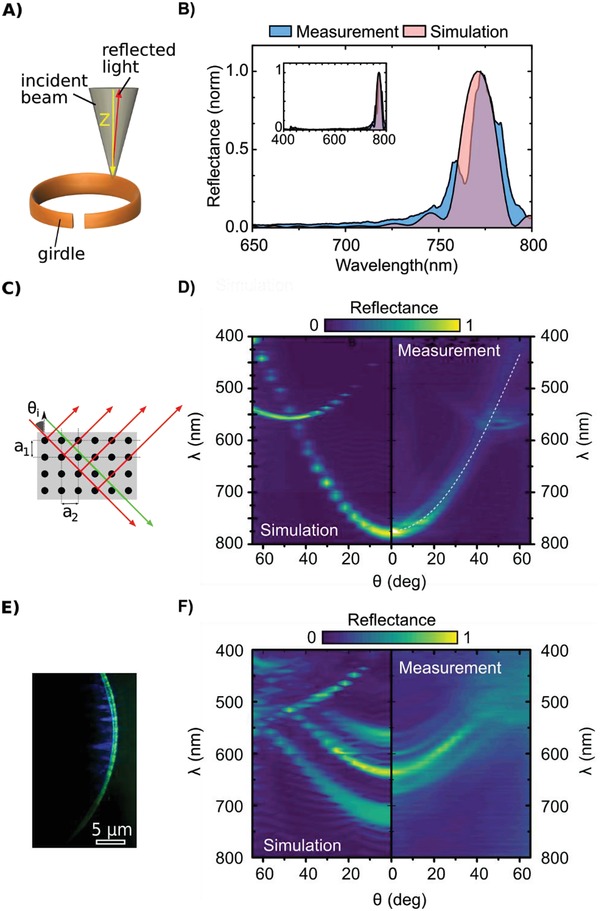
Photonic properties of the *C. granii* girdle. A) Sketch of the experimental setup, describing the direction of the focused white light (diameter ≈2 µm) on the girdle in the *Z*‐direction. B) Experimental and FDTD simulations for the reflectance at normal incidence. C) Sketch illustrating how light interferes with the micropores over periods *a*_1_ and *a*_2_ in the *Z*‐direction. D) Pseudogap and in‐plane diffraction of guided modes, shown by reflectance as a function of angle of light incidence and wavelength, observed with an oil‐immersed objective (100×) with large numerical aperture (NA = 1.45). The dashed white line represents the numerical approximation, using the lattice parameters from Figure 1F. E) RGB photograph showing waveguiding of green light as suggested by Fuhrmann et al. in ref. [16]. F) FDTD simulation and Fourier‐micro‐spectroscopy measurements of the girdle in air.

To probe the photonic properties of the girdle, we measured reflectance of a specimen lying along the *Z*‐direction normal to the glass cover slide (**Figure** **3**A). Figure 3B shows the well‐defined reflectance from a girdle immersed in water, peaking at the central wavelength (λ_c_) ≈ 780 nm (near‐infrared; NIR) at normal light incidence (see also Figure S2, Supporting Information, with absolute reflectance values). The high reflectance and well‐defined shape of the central peak demonstrate a high degree of lattice order at optical wavelength scales. The outstanding fit of the experimental measurements with finite difference time domain (FDTD) analysis does also corroborate the crystallographic quality of the lattice. The simulations were performed taking into account realistic parameters for the material (effective refractive index of nanoporous material) and morphological characteristics. The best agreement was achieved for morphological parameters from Figure 1F, the refractive indices of water (*n*_water_ = 1.33) and bulk silica (*n*_silica_ = 1.45), and considering δ_i_ = 0.05, where δ_i_ describes the void volume introduced to the silica slab by nanoporosity. By this, the resulting effective refractive index of the silica slab is *n*_silica_eff_ = 1.44 in water and *n*_silica_eff_ = 1.43 in air. We concluded that the strong central reflectance in the NIR indicated a pseudogap of the photonic bands, i.e., a photonic band gap that only exists for propagation in particular directions within the PhC structure.^[^ ^28^ ^]^

To investigate the pseudogap further, we measured reflectance as a function of light wavelength and angle of incidence (θ_i_) and fitted the measurements with the effective refractive index approximation.^[^ ^29^ ^]^ Using this approach, the 2D lattice of pores in the *Y*‐direction can be described as a Bragg stack with period *a*_1_ (Figure 3C). The contour plot in Figure 3D illustrates that the pseudogap blue‐shifted as θ_i_ increased. The dashed white line shows the conformity of the effective refractive index approach confirming the reliability of parameters introduced in the FDTD. In addition to the reflectance caused by the pseudogap, a secondary reflectance pattern appeared at λ ≈ 500 nm for θ_i_ ≈ 30°. This secondary reflectance pattern red‐shifted and increased in its reflectance intensity toward a maximum at λ ≈ 560 nm for θ_i_ ≈ 50°, where it crossed the pseudogap. The secondary reflectance pattern could be explained by in‐plane diffraction of the guided modes over period *a*_2_ in the *X*‐direction, which is indicative of the 2D symmetry of the sPhC lattice as well as a proof of the high quality of the natural lattice inspected here. To provide visual evidence of this property, we observed a water‐immersed girdle, illuminated with large angles, while collecting reflected light with large numerical aperture lens (Figure 3E). Results showed that the girdle band produced strong structural colors through in‐plane diffraction in the visible spectral range under this particular illumination conditions. Similar visual microscopic observations were communicated, but not explained, in the two earlier studies concerning the *C. granii* girdle.^[^ ^16^, ^23^ ^]^ The validity of the theoretical approximations was further tested on girdles in air (*n*_air_ = 1.00), in the same experimental configuration. As shown in Figure 3F, blue‐shifted optical features with a strong, central optical peak around λ_c_ ≈ 630 nm were observed. In addition to the central maximum, reflectance peaks occurred at λ_c_ ≈ 550, 570, and 650 nm. These peaks are due to a combination of higher‐order grating modes and internal reflections. This effect is particularly sensitive to high refractive index contrast. The FDTD simulations fitted the central maximum and showed the expected additional reflectance peaks. The qualitative mismatch between the wavelength position of simulated and measured results is likely due to additional complexities related to the sample, such as tilt or detachment from the substrate, while the simulation assumes an idealized system.

### 2.3 Phenotypic Preservation of the Girdle sPhC

**Figure 4:**
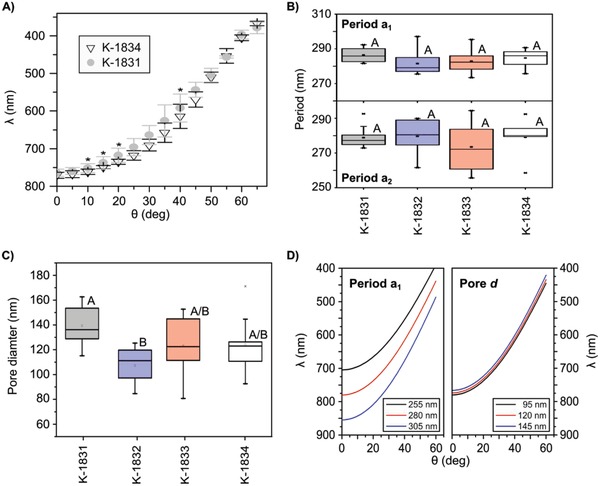
Comparison of PhC properties in different *C. granii* cell culture strains. A) Comparison of dispersion of the pseudogap in two strains of *C. granii* (K‐1831 and K‐1834) immersed in water. Significant differences, as determined with *T*‐Test, are indicated with asterisk (*) at *p* ≤ 0.05 level (*N* = 5). B) Comparison of the girdle lattice period (*a*_1_,_2_) in four strains of *C. granii*. C) Comparison of the surface micropore diameter in four strains of *C. granii*. Different capital letters in panels (B) and (C) indicate significant difference at *p* ≤ 0.05 level, tested with one factorial ANOVA and Holm Sidak posthoc tests. D) Effective refractive index approximation while varying the period of the unit cell (period *a*_1_), or the surface micropore diameter (pore *d*), respectively.

**Figure 5:**
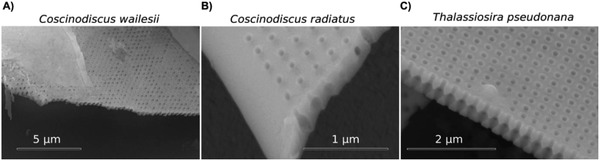
Internal girdle morphologies of different centric diatom species. All centric species tested in this study showed similar internal girdle crystallography, but varied in the period (*a*_1_) of the unit cell. A) *C. wailesii*, 332 ± 25 nm. B) *C. radiatus*, 235 ± 11 nm. C) *T. pseudonana*, 281 ± 8 nm.

To investigate the degree of preservation of the sPhC of the *C. granii* girdle, we validated the optical measurements on a second *C. granii* strain. In **Figure** **4**A, we show the high similarity of the angular dispersion of the pseudogap between the strains K‐1834 and K‐1831. A further comparison, concluded in Figure 4B, confirmed the high level of preservation for the lattice period (a_1_ and a_2_) between four different cell culture strains of *C. granii* (*a*_1_: *P* = 0.244, *a*_2_: *P* = 0.534; *N* = 10); while simultaneously, the surface pore diameter *d* (Figure 4C) could significantly differ between some cell culture strains (*P* = 0.008; *N* = 10). Using our optical model (effective refractive index approximation), we found that the pseudogap dispersion is strongly affected by variation in period *a*_1_, but nearly unaffected by variation of the pore diameter *d* (Figure 4D). We then also investigated the interspecies preservation of the sPhC morphology, by observing the girdles of three further centric diatom species using SEM techniques. All species in our experiments showed surface pores in a similar way to *C. granii* girdles. Cross‐sections furthermore confirmed similar inner morphologies, including a central chamber at the core of the unit cell (**Figure** **5**). Such internal structures were previously presented in micrographs of the species *C. wailesii* and *Thalasiosira sp*. without further morphological characterization.^[^ ^30^, ^31^ ^]^ Although experimental confirmation of sPhC behavior for these specieś girdles is pending, the high structural resemblance suggests likely comparable PhC properties as demonstrated for *C. granii*. We found, however, that the period of the unit cell varied in the range 281 ± 8, 235 ± 11, and 332 ± 25 nm between these species, i.e., *Thalasiosira pseudonana*, *C. radiatus*, and *C. wailesii*, respectively (determined on single SEM micrographs).

## 3 Discussion

To the best of our knowledge, the diatom girdle is the first known example of sPhCs entirely produced by a natural system. As well as the small number of studies on the optical properties, earlier morphological characterization have also lacked important details,^[^ ^16^, ^23^ ^]^ i.e., the internal 3D morphology and material properties of the silica slab including nanoporosity, which are essential for building the unique photonic response in the *C. granii* girdle. Using FDTD and refractive index approximations, we could also show that the girdle slab is nanoporous (δ_i_ ≈ 0.05), in addition to the volume occupied by the micropores (≈0.25–0.30) arranged in highly ordered square lattices.

Although some photonic systems in photosynthetic organisms have been described in the past few years, direct proof of their effects on photosynthesis, or on other physiological processes, remains elusive. It has been suggested that some plants and macro‐algae use photonic structures to support their light harvesting and photosynthesis.^[^ ^6^, ^7^ ^]^ Earlier studies also discussed the possible role of the frustule related to its light modulating properties for photosynthesis, mainly by facilitated passage for photosynthetically more productive, or attenuation of potentially harmful radiation.^[^ ^18^, ^32^ ^]^ As both sPhC properties (pseudogap and guided modes) resonate in the range where light absorption for photosynthesis in diatoms is low,^[^ ^33^ ^]^ we speculate that the *C. granii* girdle sPhC is not tuned for manipulation of photosynthetically more productive wavelengths. In fact, one could argue that the girdle sPhC properties are tailored around the photosynthetic action spectrum, to not interfere with pigment light absorption. We speculate that the photonic response of the girdle could also be involved in processes downstream of photosynthetic light absorption, e.g., during energy dissipation or cellular capture of heat, or may play a role in the perception of light for orientation in space (e.g., depth of the water column) or time (e.g., season of the year).

Evolutionary theory suggests that traits are preserved when they fit for a functional role they perform. Interestingly, the period of the unit cell—one important factor affecting the PhC character of the girdle slab—was highly preserved, while the pore diameter—which does not affect this behavior—varied between the different cell lines tested in our study. The high level of preservation of the photonic character between individuals and between different cell culture strains could indicate a preserved functional trait for the diatom *C. granii*. The presence of very similar crystallographic structures in the girdles of other centric diatoms (shown for the species *T. pseudonana*, *C. radiatus*, and *C. wailesii*) suggest that sPhCs are widespread within the clade of centric diatoms; therefore possibly important for the organism. A phenological trait can also occur as a spandrel, i.e., a feature without function enforced as by‐product by a functional trait. In some cases, the spandrel can evolve secondary functions over time.^[^ ^34^ ^]^ Girdle sPhC properties could have evolved as such a spandrel caused by the girdle structure, which, e.g., facilitates physical strength^[^ ^19^ ^]^ or filters harmful agents.^[^ ^21^ ^]^ However, there is to date no consensus why the periodic features of the frustule exist.

From a technical perspective, our findings show that the diatom girdle sPhCs are an example of very precise photonic nanostructure. The presence of nanoporosity in the silica slab and its high flexibility^[^ ^19^ ^]^ provide additional unique material properties. Moreover, the photonic properties are highly reproducible under controlled conditions in the laboratory which would allow production up‐scaling for technical applications. Indeed, the large variety of sPhC properties of girdles from different centric diatoms (imposed by the various periods over their girdle pores) is a very exciting finding which might in future serve as platform for a wide range of photonic innovation. The low production costs make diatom girdles cost‐effective alternatives to artificial nanofabricated sPhCs. Furthermore, diatom sPhCs can be produced with high reproducibility under conditions where modern nanofabrication techniques and cleanrooms are not available. The tuneability of diatom girdle sPhCs under different environmental conditions remains so far unknown.^[^ ^35^ ^]^ We propose that the diatom girdle can open the road for environmentally friendly, photonic‐chip‐like applications produced using natural nanostructures.

## 4 Conclusions

Our results demonstrate that diatoms fabricate sophisticated SiO_2_ sPhCs with only few requirements, fundamentally only nutrients, silicic acid, water, and light (see the Experimental Section, cultivation of diatom strains). We show that for relatively low refractive index contrast conditions (diatom immersed in water or air), the girdle supports the most characteristic features of sPhCs: a pseudogap in the NIR and in‐plane diffraction of waveguided modes in the green spectral range, as indicated by our reflectance measurements. These properties are highly reproducible in individual specimens and between different cell lines of the species *C. granii*. Other diatoms evolved similar structures with various periods, thus offering a wide range of bandgap structures for some applications. These properties could be even further enhanced by increasing the refractive index contrast between slab and surrounding media once the girdle band has been isolated. Hence, natural biomineralization of high‐end sPhCs as offered by the diatoms could become a cost‐effective alternative, when facilities or financial resources for the fabrication of artificial PhCs are missing.

## 5 Experimental Section

##### Cultivation of Diatom Strains

Culture strains of *C. granii* (K‐1831, K‐1832, K‐1833, and K‐1834) and *Thalasiosira pseudonana* (K‐1282) were purchased from the Norwegian Culture Collection of Algae (NORCCA). The species *Coscinodiscus wailesii* (CCAP 1013/9) was purchased from the Scottish Culture Collection of Algae and Protozoa (CCAP). Note that the diatom strains used in this study each originated from one single isolated cell, whereupon they proliferated by asexual and sexual reproduction during long‐term cultivation in a culture collection. Diatom cell cultures were grown in L1 diatom medium^[^ ^36^ ^]^ with a seawater base (30‰ salinity) and kept at a constant 18 °C under low white light illumination (≈30 µmol m^−2^ s^−1^).

##### Frustule Preparation and Removal of Organic Matter

Variable volumes (*V*) of diatom cultures were transferred to tubes. Initially, CaCO_3_ deposits were removed with $\frac{\text{V}}{10}$ mL of 10% HCl. Afterward, $\frac{\text{V}}{5}$ mL of 30% H_2_SO_4_ and $\frac{\text{V}}{1}$ mL of saturated potassium permanganate were added and left overnight. Then, saturated oxalic acid was added until the mixture turned transparent. The mixture was centrifuged, before the supernatant was discarded and replaced with MilliQ water. This washing step was repeated thrice. Cleaned frustules were kept in MilliQ water until investigation. The cleaning procedure was adapted from ref. [ 37 ].

##### Electron Microscopy and Morphological Analysis

Cleaned frustules were drop‐cast onto silicon wafers and left to dry at 60 °C, followed by 7 nm gold deposition with a multi‐target confocal sputtering tool (Kenosistec, Binasco, ITA). The cover slip was mounted on a microscope stub and grounded with Electrodag silver paint. Frustule surface structures of ten *C. granii* individual specimen of four strains (K‐1831, K‐1832, K‐1833, and strain K‐1834) were observed in nontilted samples with a Quanta 650 FEG SEM (FEI, Oregon, USA), or with a dual beam focused ion beam SEM (FEI, Oregon, USA). The gold‐covered frustules were shattered by pressing a glass cover slip sharply on top of the silicon wafer. By this, girdle fragments align normal to the stub with the *X*‐ and *Y*‐direction, facilitating observation of surface features in nontilted mode. At the side of fracture, the internal morphology could be studied. Structures were analyzed on SEM micrographs using Fiji.^[^ ^38^ ^]^ Dimensions were aligned with a Pelcotec Critical Dimension Standard (AISthesis Products, Inc., Clyde, USA). Periods *a*_1_ and *a*_2_ and pore diameter *d* were measured over 20 surface micropores per girdle micrograph. In total, ten individual girdles were measured for all *C. granii* strains, while one exemplary girdle was studied for each of the other centric diatom species. Measurements of internal structures were performed over five individual girdles for the species *C. granii*.

##### Reflectance Measurements with Fourier Microscatterometry

Samples were characterized on an advanced Fourier image spectroscopy in a microscope setup adapted for the study of natural photonic systems.^[^ ^7^ ^]^ Initially, glass cover slips were prepared with poly‐L‐lysine solution (P4707, Sigma Aldrich, St. Louis, USA). By this, frustule pieces were fixed onto the glass and did not move during immersion measurements with water. Frustule samples kept in water were drop‐cast on a thin glass cover slip, and measured in water, or left to dry in an oven at 60 °C for 24 h for measurements in air. A second glass cover slip was placed above of the sample using electrical tape as a spacer, to prevent large frustule pieces from breaking. White light illumination from a tungsten‐halogen lamp was coupled with a 50 µm multimode optical fiber, then collimated and focused on the sample with a high numerical aperture oil‐immersion lens (Nikon Plan‐Apochromat 100 × NA 1.45 oil OFN25). The beam was reduced to a spot diameter of ≈2 µm to probe reflectance from the girdle. Reflectance was collected for angles θ < asin(NA/*n*_oil_), with NA being the numerical aperture of the objective lens and *n*_oil_ the refractive index of the immersion medium. In this case, NA = 1.45 and *n*_oil_ = 1.51 therefore allowed for collection of θ_max_ = 74°. No movement or rotation of the sample was performed during measurement to ensure that the same volume of the sample was inspected for all angles. Reflected light was collected with a 100 µm optical fiber connected to a 2000+ Ocean Optics (Dunedin, USA) spectrometer. Each individual spectrum was normalized against the reflectance spectrum of a silver mirror measured under the same conditions. All spectral measurements were repeated on five specimens per group, if not stated otherwise.

##### Simulation and Modeling of Photonic Properties with FDTD

To model the photonic response of the girdle band, commercial implementations of FDTD technique (Lumerical FDTD Ltd, Vancouver, CAN) was used. The geometry of the internal girdle structure used for the simulations was obtained from the SEM analysis (Figure 1F). The selection of the refractive index parameters of the simulated structure considered a nanoporous nature of the material of the girdle, and is defined below.

##### Approximation of Nanoporosity

A table with abbreviations used for the refractive index approximations is provided in Table S1 in the Supporting Information. The silica forming the frustule was assumed to be composed of SiO_2_ nanoparticles packed within a volume. The void between nanoparticles was filled with the surrounding medium of refractive index (*n*_i_). Therefore, the refractive index $\;n_{\text{silica}\_\text{eff}}$ needed to be defined according to nanoporosity and fraction of volume occupied by the surrounding media of the biosilica (δ_i_). The Maxwell–Garnett approximation was used,^[^ ^39^ ^]^ which was validated for other natural nanoporous photonic structures.^[^ ^27^ ^]^ This model showed the same results as more complex approximations (e.g., Bruggeman model) considering the low refractive index contrast and low porosity of the diatom biosilica in water or air. Hence, the effective dielectric constant of the biosilica ($\varepsilon_{\text{silica}\_\text{eff}}=n_{\text{silica}\_\text{eff}}^{2}$) could be calculated, as

(1) $$\left(\frac{\varepsilon_{\text{silica}\_\text{eff}}-\varepsilon_{\text{silica}}}{\varepsilon_{\text{silica}\_\text{eff}}+2\varepsilon_{\text{silica}}}\right)=\delta_{\text{i}}\;\left(\frac{\varepsilon_{\text{i}}-\varepsilon_{\text{silica}}}{\varepsilon_{\text{i}}+2\varepsilon_{\text{silica}}}\right)$$

where $\;\varepsilon_{\text{silica}}=n_{\text{silica}}^{2}$ is the dielectric constant of bulk nonporous silica (*n*_silica_ = 1.45), ε_i_ = *n*_i_^2^ is the dielectric constant of the immersion material, and δ_i_ is the nanoporosity value (0 < δ_i_ < 1). *n*_i_ = 1.00 and 1.33 were considered depending on whether the girdle was immersed in air or water, respectively. The nanoporosity value was determined by iteration of δ_i_ in the FDTD reflectance calculation, until a maximum fit was achieved. By this, obtained was δ_i_ ≈ 0.05, which was corresponded to effective refractive indices of *n*_silica_eff_ = 1.43 and 1.44 for a girdle band in air and water, respectively.

##### Effective Refractive Index Approximation for Micropores

For the Bragg scattering approximation shown in Figure 4D,F, the effective refractive index approximation was used, validated previously for 2D heterostructure sPhCs.^[^ ^29^ ^]^ Each of the unit cells was considered as a scattering point arranged in the *XZ* plane (Figure 3C). Applying Bragg's law,

(2) $$\lambda_{\text{c}}=2a_{1}{\left(n_{\text{c}}^{2}-n_{\text{i}}^{2}{\text{sin}}^{2}\theta_{\text{in}}\right)}^{1/2}$$

where λ_c_ is the central wavelength of the reflectance peak, *a*_1_ is the distance between planes which corresponds to the modulus of the lattice vector, θ_in_ is the incident angle respective to the normal plane of the PhCs, and ε_c _ = $n_{\text{c}}^{2}\;$ is the effective dielectric constants of the PhC, which has to be differentiated from the effective refractive index of the biosilica (*n*_silica_), as calculated before. This approximation relied on the effective refractive index of the PhC slab (*n*_c_) induced by the micropore void. Previous studies on complex 3D PhCs defined the effective refractive index of a PhC, by taking into account the void filling fraction for a given material inside the unit cell.^[^ ^40^ ^]^ In the case of the girdle, the void filling fraction (*f*_i_) of the unit cell could be calculated as

(3) $$f_{\text{i}}=\frac{V_{\text{DC}\;}+\;3V_{\text{p}}}{V_{\text{t}}}\;=\;\frac{\pi }{Da_{1}a_{2}}\left[\frac{C^{3}}{6}+\frac{3}{2}hd^{2}\right]$$

where *V*_DC_ and *V*_p_ are the volumes of the double cone chamber and the *X*‐, *Y*‐, and *Z*‐pores, respectively. Note that the interconnection of chambers was considered by cylinders in the *X*‐ and the *Z*‐direction with the same diameter, but, considering that *C* > *h*_1,2_ (Figure 1E), the double cone chamber occupied the space of the *X*‐ and the *Z*‐axis pores. All volumes were filled by the immersion medium (*n*_i_). The remaining volume was filled with silica (*n*_silica_eff_). *V*_t_ is the total volume of the unit cell with dimensions D and *a*_1,2_. Note that a perfect square lattice (*a*_1,2_ = *a*), the *X*‐, *Y*‐, and *Z*‐pores with same dimensions, and the double cone chamber as a sphere for the sake of simplification were considered. By this, the effective refractive index of the PhC can be defined as:

(4) $$n_{\text{c}}=\;{\left(f_{\text{i}}n_{\text{i}}^{2}+\left(1-f_{\text{i}}\right)n_{\text{silica}\_\text{eff}}^{2}\right)}^{1/2}$$

With this approximation we obtained that *n*_c_ = 1.25 and 1.39 for a girdle in air and water, respectively. Note that these values were dependent on *n*_i_, on which also *n*_silica_eff_ was depended.

##### Statistical Analysis

Lattice vectors *a*_1,2_ in the *C. granii* strain K‐1834 were tested with one‐factorial analysis of variance (ANOVA) followed by Holm Sidak posthoc analysis. Comparison of four *C. granii* cell culture strains concerning differences in period *a*_1_, *a*_2_ and pore diameter *d* were treated in the same way. Reflectance data of K‐1831 and K‐1832 girdles were tested with one‐tail, homoscedastic *T*‐test at increments of 5° theta. Differences at *p* > 0.05 level were considered significant. Statistics were performed using the software Origin (OriginLab Corporation, Northampton, USA).

## Conflict of Interest

The authors declare no conflict of interest.

## Supporting information

Supporting Information
