# Supplementary material for: Highly Reproducible, Bio‐Based Slab Photonic Crystals Grown by Diatoms
Source: Adv Sci (Weinh). 2020 Mar 21;7(10):1903726. doi: 10.1002/advs.201903726 (PMC7237861; doi:10.1002/advs.201903726)
Supplement: Supplementary file 1 — Supporting Information [file ADVS-7-1903726-s001.pdf]

Copyright WILEY-VCH Verlag GmbH & Co. KGaA, 69469 Weinheim, Germany, 2020.

ADVANCED SCIENCE
Open Access

Supporting Information

for *Adv. Sci.,* DOI: 10.1002/advs.201903726

Highly Reproducible, Bio-Based Slab Photonic Crystals Grown by Diatoms

Johannes W. Goessling, William P. Wardley, and Martin Lopez-Garcia**

Copyright WILEY-VCH Verlag GmbH & Co. KGaA, 69469 Weinheim, Germany, 2020.

Supporting Information

Highly reproducible, bio-based slab photonic crystals grown by diatoms

Johannes W. Goessling, William P. Wardley, Martin Lopez Garcia**

**corresponding authors*

Table S1: Abbreviations used for morphological lattice description and refractive index approximations

δ_i	Filling volume of the immersion media in the nanoporosity approximation
f_i	Void filling fraction of the micropores
n_i	Refractive index of the surrounding medium
n_{air}	Refractive index of air (=1.00)
n_{water}	Refractive index of water (=1.33)
n_{silica}	Refractive index of the bulk silica material
n_{silica_eff}	Effective refractive index of the silica slab including nanoporosity (δ_i)
n_c	Effective refractive index of the PhC
ε_{bulk}	Effective dielectric constant of the bulk silica material
ε_{silica}	Effective dielectric constant of the silica slab considering nanoporosity
ε_i	Effective dielectric constant of the surrounding medium
ε_c	Effective dielectric constant of the PhC

Photonic bands of the girdle

Although a full description in terms of photonic bands is outside of the scope of this work, we present, as supplementary information, the photonic band calculation using the same simulation parameters described in Fig. 1F of the manuscript. We therefore consider a_1=285 and a_2=278 in this case with d= 100 nm. To calculate the photonic bands of the girdles in water, we used the plane wave expansion method implementation provided by the MPB software [12]. Since we were mostly interested in the description of totally confined modes (the ones inspected in our experimental configuration) we can approximate the lattice as pure 2D system instead of a 2.5D photonic crystal. Therefore, the calculation of the

photonic bands is performed considering silica material perforated by a 2D lattice of infinite rods filled by refractive index n_i = 1.33. For the silica, we used the values provided by the Maxwell-Garnett approximation of the silica (see methods in the main text). Under this approximation n_c = 1.44. As can be observed, the photonic bands in Fig. S2 shows the low energy pseudogap for λ≈785nm, which is the same spectral range for which we report the experimental evidence in this work (see Fig. 3).

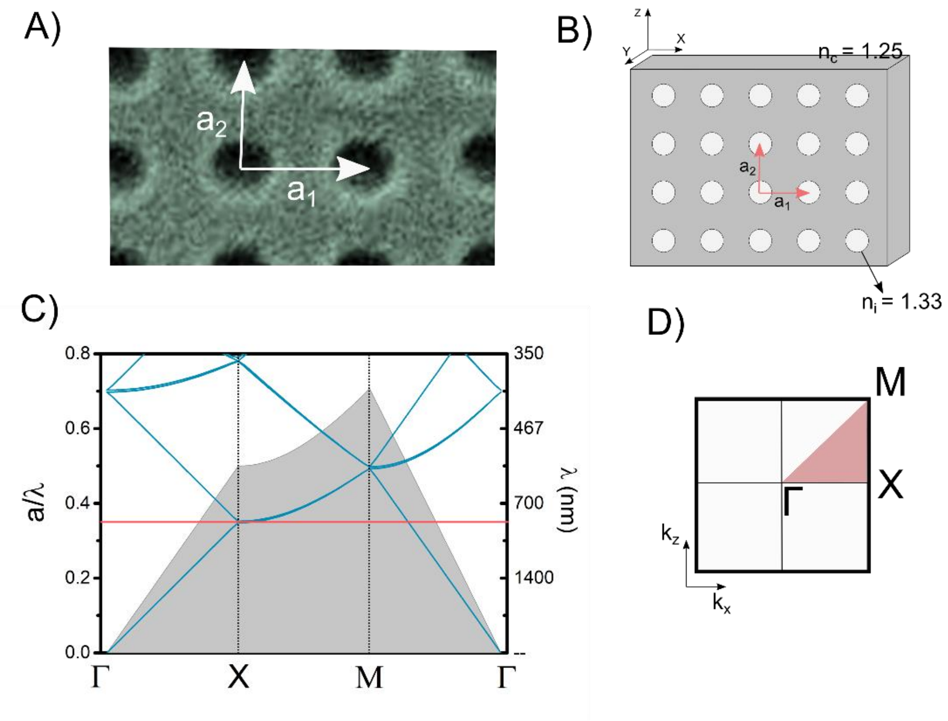

Figure S1: A) Lattice vectors as defined over an SEM image of the girdle band photonic structure. B) Sketch of the lattice and refractive indices for bulk material (n_c = 1.44) and rod filling material (n_i = 1.33). C) Photonic bands calculation for TM modes. Solid red horizontal line indicates spectral position of the pseudogap. D) Brillouin zone for a square lattice.

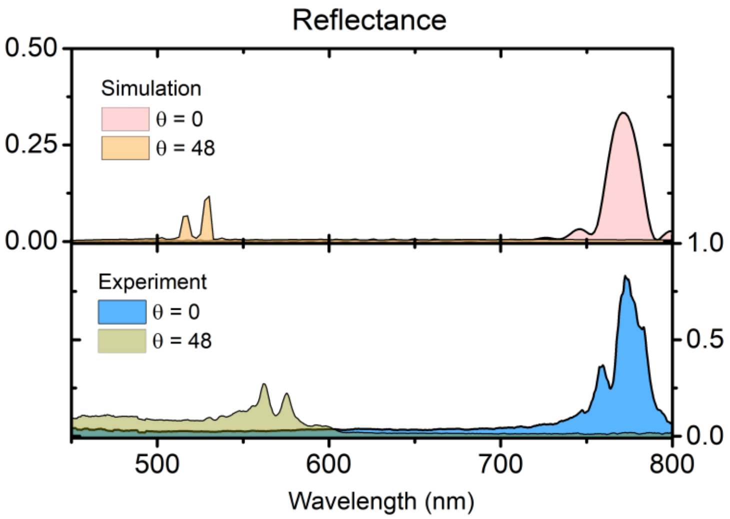

Figure S2: Single absolute reflectance spectrum for two different incident angles and for the girdle in water shown in Fig. 3D. Note that the values shown in the main text have been normalized to the maximum reflectance. The experimental absolute values shown here are the result of normalization to a silver mirror spectrum measured in the same conditions than the samples (see methods). The experimental values present larger values of reflectance than the predicted simulation, due to the complex illumination necessary to inspect the girdle in the appropriate lattice direction. However, the spectral shape of simulation and measurement are on a very good agreement both angles regardless of a slight detuning for θ = 48 deg.
